# ФC31 Integrase-Mediated Isolation and Characterization of Novel Safe Harbors for Transgene Expression in the Pig Genome

**DOI:** 10.3390/ijms19010149

**Published:** 2018-01-04

**Authors:** Yanzhen Bi, Zaidong Hua, Hongyan Ren, Liping Zhang, Hongwei Xiao, Ximei Liu, Wenjun Hua, Shuqi Mei, Adrian Molenaar, Götz Laible, Xinmin Zheng

**Affiliations:** 1 Hubei Key Laboratory of Animal Embryo Engineering and Molecular Breeding, Hubei Institute of Animal Science and Veterinary Medicine, Hubei Academy of AgroSciences, Wuhan 430064, China; zaidonghua@163.com (Z.H.); renhongyan507@163.com (H.R.); chzlp1982@163.com (L.Z.); xiaohongwei2003@163.com (H.X.); anbit20@hotmail.com (X.L.); huawenjun08@aliyun.com (W.H.); msqlfe@163.com (S.M.); 2 AgResearch Ltd., Grasslands Research Centre, Private Bag 11008, Palmerston North 4442, New Zealand; adrian.molenaar@agresearch.co.nz; 3 AgResearch Ltd., Ruakura Research Centre, Private Bag 3123, Hamilton 3214, New Zealand; goetz.laible@agresearch.co.nz

**Keywords:** ФC31 integrase, pseudo attP site, safe harbor, transgene, pig

## Abstract

Programmable nucleases have allowed the rapid development of gene editing and transgenics, but the technology still suffers from the lack of predefined genetic loci for reliable transgene expression and maintenance. To address this issue, we used ФC31 integrase to navigate the porcine genome and identify the pseudo attP sites suitable as safe harbors for sustained transgene expression. The combined ФC31 integrase mRNA and an enhanced green fluorescence protein (EGFP) reporter donor were microinjected into one-cell zygotes for transgene integration. Among the resulting seven EGFP-positive piglets, two had transgene integrations at pseudo attP sites, located in an intergenic region of chromosome 1 (chr1-attP) and the 6th intron of the *TRABD2A* gene on chromosome 3 (chr3-attP), respectively. The integration structure was determined by TAIL-PCR and Southern blotting. Primary fibroblast cells were isolated from the two piglets and examined using fluorescence-activated cell sorting (FACS) and enzyme-linked immunosorbent assay (ELISA), which demonstrated that the chr1-attP site was more potent than chr3-attP site in supporting the EGFP expression. Both piglets had green feet under the emission of UV light, and pelleted primary fibroblast cells were green-colored under natural light, corroborating that the two pseudo attP sites are beneficial to transgene expression. The discovery of these two novel safe harbors for robust and durable transgene expression will greatly facilitate the use of transgenic pigs for basic, biomedical and agricultural studies and applications.

## 1. Introduction

Programmable nucleases like Clustered regularly interspaced short palindromic repeats/Cas9 (CRSIPR/Cas9), transcription activator-like effector nuclease (TALEn) and Zinc-finger nucleases (ZFN) have made considerable contributions to the development of precision gene editing and transgenics, but up to date they still face limitations due to a shortage of known sites for reliable and sustained transgene expression in transgenic livestock [1]. The pig in particular, represents an important animal model with significance to both agriculture and biomedicine, yet the predefined genetic loci available for robust and durable transgene expression within the pig genome are very limited. To date only a handful of such loci in the pig have been studied, informed by comparative genomics of rodents, i.e., Rosa26, H11 and *COL1A* sites [2,3,4,5]. There is an ever-increasing need to identify novel safe harbors in the pig genome so as to expand the toolbox to generate transgenic pigs for robust and site-specific transgene expression for economic trait improvement, disease modeling and mass production of therapeutic proteins [6]. But up to now there has been no powerful approach available to identify novel safe sites in vivo at genome-wide level.

To address this issue, we took advantage of ФC31 integrase to integrate a reporter gene into pseudo attP sites of the pig genome so as to assess the expression potential of these sites. As shown in previous work [7], ФC31 integrase is capable of catalyzing site-specific DNA recombination between an attB-containing donor plasmid and a pseudo attP site in mammalian genomes to form a single-copy and unidirectional genetic structure, which is believed to be beneficial to transgene expression [8,9,10]. More importantly, the profiling of the pseudo attP sites in a given mammalian genome would help determine candidate sites for transgene integration and expression [11]. Therefore, ФC31 integrase can be harnessed to locate potential safe harbors in the pig genome. Here, we isolated two novel pseudo attP sites in the pig genome using ФC31 integrase and characterized them as safe harbors for site-specific transgene integration and robust transgene expression. These sites add to our repertoire and provide new options to stably integrate a wide range of transgenes for robust and predictable expression. We envision that these novel safe harbors will facilitate the creation of transgenic pigs for agricultural and biomedical applications.

## 2. Results

### 2.1. The ФC31 Integrase is Functional in Catalyzing Site-Specific Recombination in Porcine Embryos

**Figure 1:**
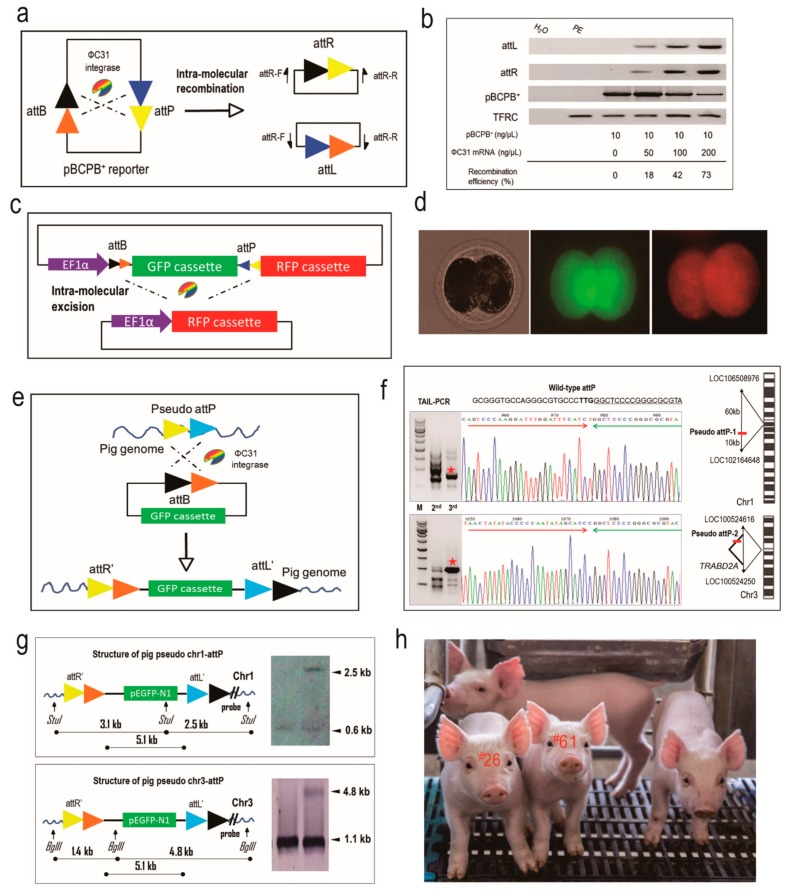
Creation of transgenic pigs mediated by ФC31 integrase-catalyzed site-specific recombination. (**a**) The catalyzing principle of intra-molecular recombination between *cis*-positioned attP and attB sites mediated by ФC31 integrase. The pBCPB^+^ plasmid would give rise to two independent smaller plasmids due to the recombination, resulting in two novel hybrid sites, attR and attL; (**b**) Recombination efficiency of ФC31 integrase in the porcine embryos. End-point PCR was performed to examine the production of hybrid sites and quantified to calculate the efficiency. The *TFRC* gene was used as an internal control. Gradient microinjection dosages are shown; (**c**) The catalyzing principle of intra-molecular recombination between trans-positioned attP and attB sites mediated by ФC31 integrase. The sequence (GFP cassette) between trans-positioned attP and attB sites will be excised and the newly joined RFP cassette will be activated; (**d**) Activation of RFP in the porcine embryos by the co-injection of the ФC31 integrase mRNA and the reporter plasmid. Left, bright field. Middle, green fluorescence field. Right, red fluorescence field; (**e**) The recombination principle of attB and pseudo attP sites in mammalian cells mediated by ФC31 integrase. A plasmid containing attB will integrate into the host genome at the pseudo attP site, creating the attR’ and attL’ sites, which forms the unidirectional and single-copy transgenic structure; (**f**) Isolation of the integration sites in the newborn piglets using TAIL-PCR. Two pseudo attP sites were identified and mapped to the given locus in the pig genome; a star denotes the TAIL-PCR product; red arrow denotes the attB sequence and green arrow denotes the pseudo attP sequence; red bar denotes the location of pseudo attP site; colored letters denote different nucleotide bases; (**g**) Southern blotting was conducted to validate the recombination at the two pseudo attP sites. Specific bands are indicated by arrows; (**h**) The image of the transgenic piglets. Piglet numbers ^#^26 and ^#^61 were the two produced by the ФC31 integrase-mediated site-specific integration.

We had shown previously that ФC31 integrase is able to induce site-specific DNA recombination in porcine somatic cells [12]. In order to test if ФC31 integrase is functional in porcine embryos, we performed two lines of tests to detect the site-specific recombination event. First, we used the episomal plasmid pBCPB^+^ to detect the intra-molecular recombination with *cis*-oriented attB and attP in porcine parthenogenetic embryos [7] (Figure 1a). A range of different amounts of ФC31 integrase mRNA combined with pBCPB^+^ plasmid were microinjected into the cytoplasm of porcine parthenogenetic embryos and the recombined attR and attL DNA sequences were detected by embryo-based PCR 48 h post microinjection when cleaved into 2-cell stage. The PCR demonstrated that the expected DNA products were amplified from all the three treatment groups, and that the combination of ФC31 integrase (200 ng/μL) and pBCPB^+^ plasmid (10 ng/μL) gave rise to the highest recombination efficiency (73% vs. 42% and 18%; Figure 1b). This result demonstrated that ФC31 integrase is dosage-dependently active in porcine embryos.

Next, we took advantage of a visual reporter plasmid to corroborate the recombination efficiency induced by ФC31 integrase mRNA in porcine embryonic environment. The pT2Kmin-XIpGbR plasmid is composed of a GFP cassette flanked by counter-oriented attB and attP sites next to a DsRed reporter [13]. In this case, the ФC31 integrase would catalyze the excision of attB and attP sites, cutting out the GFP cassette and activating DsRed expression (Figure 1c). Twenty four hours of microinjection of ФC31 integrase mRNA and pT2Kmin-XIpGbR plasmid into the porcine parthenogenetic embryos, these embryos were examined with a fluorescence microscope for DsRed expression. As shown in Figure 1d, red fluorescence could be observed in the embryos treated with both ФC31 integrase mRNA and pT2Kmin-XIpGbR, but not in the embryos injected with only the pT2Kmin-XIpGbR plasmid. This assay further proved the functionality of ФC31 integrase mRNA in the porcine embryonic environment, forming the basis for a microinjection trial for ФC31 integrase-mediated transgene integration in porcine zygotes.

### 2.2. The Integration of a Reporter Donor into Pseudo attP Sites Using ФC31 Integrase by Zygote Microinjection

We then attempted to utilize ФC31 integrase to site-specifically integrate a transgene into the pig genome. As found in previous studies, ФC31 integrase is capable of recognizing pseudo attP sites in mammalian genomes, catalyzing the recombination between the attB site in the donor DNA and the pseudo attP site, resulting in an unidirectional and single-copy transgenic structure [8] (Figure 1e). For successful integration of donor DNA into the pig genome to occur, ФC31 integrase mRNA needs to be translated in the cytoplasm and transported into nucleus, with the donor DNA available in the nucleus as integration substrate. Two routes were adopted to achieve that end, i.e., intracytoplasmic microinjection (ICI) and pronuclear microinjection (PNI) of ФC31 integrase mRNA and donor DNA into porcine zygotes. The dosage was consistent with that optimized in parthenogenetic embryos. For ICI, a total of 59 one cell-stage embryos were microinjected and 47 of them were transferred into two surrogate sows. One surrogate returned, while the other gave birth to seven piglets. However, no piglets positive for EGFP integration were detected among these seven piglets.

For PNI, a total of 93 one cell-stage embryos were microinjected, and 73 of them were transferred into three surrogate pigs. This resulted in the birth of 25 live piglets, seven of which had an integrated EGFP reporter, as demonstrated by end-point PCR and dot blot (Table 1, and Figures S1 and S2).

In order to test if the donor DNA integration was mediated by ФC31 integrase, we performed TAIL-PCR to identify the integration sites from the seven EGFP-integrated piglets. We were able to amplify specific genomic DNA fragments from two piglets (^#^26 and ^#^61), while TAIL-PCR for the other five piglets generated smear products and failed to amplify specific DNA fragments. The specific TAIL-PCR products generated from the two piglets (^#^26 and ^#^61) were sequenced and aligned to the wild-type attP sites. This revealed that the two piglets had the EGFP transgene integrated at pseudo attP sites, which confirmed earlier results in porcine somatic cells (Figure 1f) [12]. The pseudo attP site of ^#^26 piglet is located in an intergenic region on chromosome 1 (abbreviated as chr1-attP), and the one in ^#^61 piglet was located in the 6th intron of the *TRABD2A* gene on chromosome 3 (abbreviated as chr3-attP), respectively. Southern blotting demonstrated that the transgenic structures of the two pseudo attP sites were single-copy, unidirectional and heterozygous, consistent with the principle of ФC31 integrase-catalyzed site-specific recombination (Figure 1g,h).

### 2.3. The Two Pseudo attP Sites Are Safe Harbors for Transgene Expression

**Figure 2:**
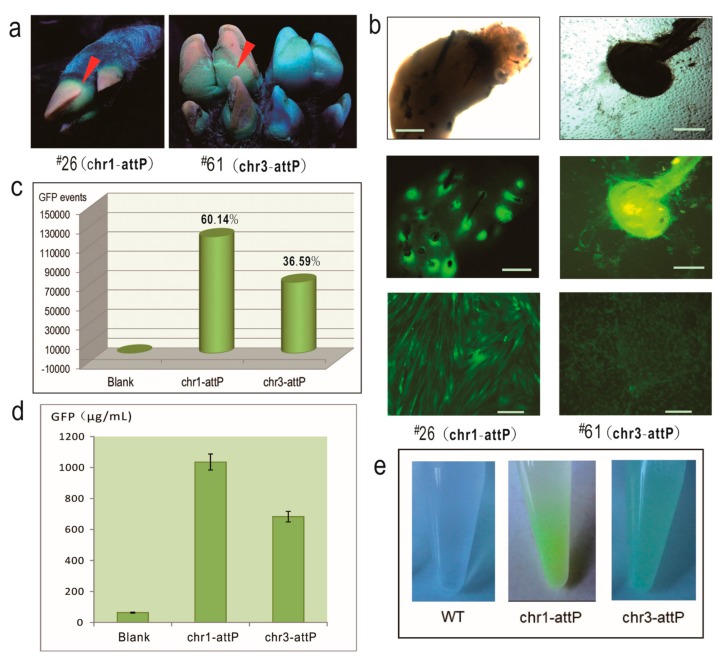
Characterization of porcine pseudo attP sites as safe harbors. (**a**) Image of the feet of EGFP-positive piglets. Red arrows indicate green fluorescence; next to ^#^61 is a control foot from a wild-type pig; (**b**) Isolation of primary fibroblast cells from the ear tissues and sorted GFP cells. From top to bottom: the images of hair follicles under bright field, hair follicles under fluorescence field, and sorted GFP cells under fluorescence field; scale bar, 10 μm; (**c**) FACS analysis showing the percentage of GFP-positive cells in the population of the primary cells isolated from EGFP-positive piglets; (**d**) The intracellular GFP concentration detected by ELISA; (**e**) Green appearance of sorted GFP-positive cells under natural light when collected in DPBS solution. WT, wild-type.

Several lines of experiments were carried out to characterize the ФC31 integrase-mediated, EGFP-integrated piglets. Firstly, EGFP expression at the individual-level was observed under UV light. As shown in Figure 2a, EGFP could be seen in the feet of the two piglets. Secondly, we isolated primary fibroblast cells from the ear tissues of the two piglets. During the first two days of the primary cell culture, bright EGFP was observed at the root of the hair follicles (Figure 2b). Once the primary fibroblast cells were established in the culture, cells were harvested for FACS to analyze the EGFP-positive cells. As shown in the Figure 2c, chr1-attP gave rise to a greater number of EGFP-positive cells than chr3-attP (120,285 ± 1312 cells vs. 73,189 ± 875 cells; 60.14 ± 6.27% vs. 36.59 ± 7.13%). As reflected again in the bottom images of Figure 2b, sorted EGFP-positive cells from chr1-attP had a brighter EGFP fluorescence than chr3-attP. Thirdly, in order to quantify the EGFP expression level in the fibroblast cells, ELISA was conducted to measure the EGFP concentration in the cell lysates of EGFP-positive cells. As shown in the Figure 2d, the EGFP concentration of chr1-attP and chr3-attP was 1035 ± 87 and 683 ± 56 μg/mL, respectively. Fourthly, when we harvested the fibroblast cells into tubes, the cells were visibly green under natural light (Figure 2e), which implies that the two pseudo attP sites are capable of supporting robust expression of integrated EGFP.

## 3. Discussion

Transgenic pigs hold promise for applications in the fields of agriculture and medicine. Tremendous effort has been invested in modifying and altering functional genes within the porcine genome to produce various lines of transgenic pigs. Nonetheless, the intricate interplays between exogenously integrated DNA sequences and the endogenous elements of the porcine chromosomal locus that can affect the phenotypes restrict the reliability of swine transgenics for basic, medical and agricultural applications. Although precision gene editing has made considerable progress, the issue of where to integrate a transgene into the porcine genome to minimize the risk of transgene expression silencing and maximize its expression efficacy has received little attention. Addressing this question would greatly accelerate the study and application of transgenic pigs.

In the pig, only a handful of studies have investigated the utility of just a few genomic loci for transgene expression, deduced from evolutionary comparative analysis. The Rosa26 locus was widely used in rodents, rats and human cells for transgene integration, and the porcine Rosa26 locus was identified recently [2]. Another porcine transgene safe harbor was H11, which was identified as the orthologue to the mouse H11 site and proved to be a reliable locus for ectopic gene expression [3]. The *COL1A* locus was recently utilized for gene knock in in pigs [4,5]. However, all three loci are located near endogenous genes that can possibly be disregulated by the addition of a transgene and thus their expression efficacy and safety profiles remain uncertain. As a result, the identification of novel porcine safe harbors is highly desirable to provide suitable solutions for the specific requirements of different transgenic applications.

To date, there have been no specialized tools developed to identify safe harbors in the mammalian genome. The ФC31 integrase, which originates from a Streptomyces phage, has been utilized to integrate transgenes into the genomes of a variety of eukaryotes, ranging from yeast to human. The integrase is capable of recognizing the pseudo attP sites in the mammalian genomes and integrating a single-copy and unidirectional transgene into the pseudo attP sites, resulting in a transgenic structure more favorable for efficient transgene expression, than when compared to random integration [14]. Accordingly, this advantage enables the direct comparison of the transgene expression efficacy at different pseudo attP sites and identifies the ideal integration locus as a safe harbor. In fact, ФC31 integrase has been applied to identify safe harbors from several mammalian species. In the bovine genome, Ou et al. discovered a safe harbor named BF4 at 4q31, where the transgene expression was as high as ~328 μg/mL, more than twice that of the other pseudo attP site [15]. Olivares et al. integrated *Factor IX* gene into the mouse genome by ФC31 integrase and identified mpsL1 as a preferential safe harbor which does not lie within coding sequences and which supports the *Factor IX* ectopic expression as high as ~4 μg/mL in the serum [8]. Bi et al. identified four pseudo attP sites in the porcine genome from cultured porcine cells and experimentally analyzed their expression efficacy in a reporter system, two of which were more potent than the other pseudo attP sites [12]. However, many these studies merely detected pseudo attP sites in cultured cell lines, which still need further research to test their availability and in vivo potential for transgene expression.

Here we extended the research to porcine fertilized embryos and identified safe harbors in the pig genome. Two microinjection routes were tested and assessed for their integration efficiency. For ICI, we failed to produce EGFP-positive piglets. We reasoned that the reporter DNA in the cytoplasm might not be efficiently transported into the nucleus for integration. For PNI, seven piglets were demonstrated to contain EGFP reporter, and two of them were identified to have EGFP integrated at pseudo attP sites. We speculated that for the other five piglets, integration was not mediated by ФC31 integrase but occurred through random integration by pronuclear microinjection. The two pseudo attP sites identified in this study recapitulated the previous work [12], demonstrating that both the two pseudo attP sites are hot spots for transgene integration and expression. In particular, chr1-attP is an ideal transgene safe harbor as it is located in an intergenic region, with very low probability for any interference with endogenous gene expression. In contrast, chr3-attP is located in the intron of an endogenous gene, which could result in potential adverse effects on the physiology of the transgenic pigs. But due to the fact that the piglets grow and feed normally and are healthy, we conclude that chr3-attP may still be considered as safe harbor for reliable transgene expression in pigs.

We also observed that only a proportion of the cells we derived from our piglets were GFP-positive. This indicated that both EGFP-positive piglets were mosaic animals and that the integrase-mediated integration must have occurred not at the one-cell stage but when the embryo had already undergone the first cleavage division. The observed range for EGFP-positive cell populations further indicates that there could be a broad time window for the activation of ФC31 integrase in individual injected embryos. Injection of the integrase as a recombinant protein might allow an immediate and narrower time window for integration events following injection and minimize the level of mosaicism.

Although CRISPR/Cas9 technology is a promising tool for the editing of mammalian genomes, applications are presently limited due to a shortage of well characterized safe harbor sites and the low efficiencies for knockins of transgenes with the CRISPR technology. Our study identified novel safe harbors in the pig genome that can be utilized to allow site-specific integration and knockin of transgenes for reliable expression while not interfering with endogenous gene functions. We also demonstrated the utility of the ФC31 integrase for the efficient knockin of transgenes in pig, implying that it is possible to directly use it in other species. Considering the advantage of ФC31 integrase-catalyzing recombination, it could be modified to further improve the gene delivery efficiency by re-targeting or repeated use of pseudo attP sites. For the upcoming studies of efficient and repeated access of these safe harbor sites, our strategy would be extended from knockin of reporter plasmid to the knockin of an attP site or recombination sites such as loxP or FRT to fully harness the enzymatic efficiencies of ФC31 integrase or recombinases. This manipulation will also make most use of the potential of these porcine pseudo attP sites to allow for robust transgene expression.

## 4. Materials and Methods

### 4.1. Animal Ethics

All experimental procedures involving animals were reviewed, approved and supervised by the Animal Care Committee of the Institute of Animal Science and Veterinary Medicine, Hubei Academy of AgroSciences, Wuhan, China (Ethics code: 2013-14. Approved date: 8 October 2013). All wild-type and EGFP-positive pigs were raised with same diet and under identical conditions.

### 4.2. Plasmids and Strains

The ФC31 integrase expression plasmid (pCMV-Int) and reporter pBCPB^+^ were obtained from Addgene (Cambridge, MA, USA). The reporter donor pEGFP-N1-attB was constructed as previously described [10]. The reporter plasmid pT2Kmin-XIpGbR was kindly provided by Dr. James A. Lister (Virginia Commonwealth University, Richmond, VA, USA). TA cloning plasmid was purchased from Takara (Dalian, China). Competent *E. coli* DH5α cells were prepared according to standard protocols. PureLink^®^ HiPure Plasmid Filter Purification Kits were used for midi and maxi preparation of all plasmid DNA (Invitrogen, Carlsbad, CA, USA). A P-class nanophotometer was used to measure the quality and quantity of DNA (Implen, Munich, Germany).

### 4.3. Nucleic Acid Manipulation

Pig genomic DNA was extracted by a Purelink Genomic DNA Mini kit (Invitrogen) and quantified using P-class nanophotometer. Probes used for Southern blotting were generated by PCR primer pairs PPP1-F/R and PPP2-F/R (Table S1) and labeled using a DIG High Prime DNA Labeling and Detection Starter Kit I (Roche, Basel, Switzerland) in accordance with the manufacturer’s instructions. For Southern blotting, 20 μg of genomic DNA was digested by StuI or BglII restriction enzymes and the resultant fragments were separated by electrophoresis in an 0.8% agarose gel run at 20 V overnight. The gel was soaked in alkali solution (0.5 M NaOH, 1.5 M NaCl) for 2 × 15 min and the denatured DNA was transferred to a nylon membrane (Amersham, Piscataway, NJ, USA) using the sandwich method [16]. The DNA was fixed by baking at 120 °C for 30 min. Hybridization was carried out at 42 °C overnight in transfer buffer (0.5 M NaOH, 1.5 M NaCl). The membrane was washed with 2 × Saline Sodium Citrate (SSC)/0.5% Sodium dodecyl sulfate (SDS) washing buffer to remove the non-specifically bound probes. Hybridization signals were detected by color development using Nitro-Blue-Tetrazolium/5-Bromo-4-Chloro-3-Indolyl Phosphate (NBT/BCIP) solution included in the kit.

The DNA sequence encoding the ФC31 integrase mRNA was amplified using PCR with T7-promoter-containing primers (Table S1) and its mRNA was in vitro synthesized using a mMESSAGE mMACHINE mRNA transcription synthesis kit (Ambion, Austin, TX, USA). The ФC31 integrase mRNA was purified using phenol:chloroform extraction and isopropanol precipitation, and resuspended in RNase-free water.

### 4.4. Pig Embryo Engineering and Microinjection

For preparation of porcine parthenogenetic embryos, selected cumulus oocyte complexes (COCs) were washed three times in Dulbecco’s Phosphate Buffered Saline (DPBS) supplemented with 5% fetal bovine serum and three times in maturation medium consisting of Medium 199 (GIBCO) supplemented with 10% (*v*/*v*) pig follicular fluid, 0.1% (*w*/*v*) polyvinyl alcohol, 3.05 mM glucose, 0.91 mM sodium pyruvate, 0.57 mM l-Cysteine, 100 IU/mL streptomycin sulphate (GIBCO), 100 IU/mL potassium penicillin G (GIBCO), 10 IU/mL PMSG (Ningbo Second Hormone Factory, Ningbo, China) and 10 IU/mL hCG (Ningbo Second Hormone Factory) [17]. The COCs were incubated for 42–44 h in a 5-well dish at 39 °C in the Submarine Incubation System with 100% humidity and 5% CO_2_. After the incubation, oocytes were denuded from cumulus cells by gent pipetting in DPBS with hyaluronidase (1 mg/mL). Oocytes with polar body I (pb I) were selected and washed three times with activation solution (0.3 M mannitol, 1 mM CaCl_2_, 0.5 mM MgSO_4_ and 0.05 mg/mL bovine serum albumin), then activated by a single DC pulse of 1.5 kV/cm for 30 μ s using a BTX Electro-Cell Manipulator 2001 (BTX Inc., San Diego, CA, USA).

The estrus conditions of all the sows (Landrace breed) were observed for 40 days (two estrus cycles) before beginning with the injection experiment. Sows (*n* = 89) exhibiting normal estrus cycles were used for surgery and embryo transfer (Figure S3). Sows showing signs of estrus were artificially inseminated twice with Large White boar semen (2 billion spermatozoa in 50 mL of semen extender) with an interval of 12 h between inseminations. Twelve hours after the second insemination, sows were anesthetized by 2.5% pentobarbital. Both of their oviducts were surgically exposed and in vivo fertilized embryos were flushed out into DPBS solution using a syringe of a 12-gauge needle. One-cell stage embryos were centrifuged in DPBS at 400× *g* for 5 min to expose the pronucleus, and then microinjected, either into the pronucleus or the cytoplasm, with the ФC31 integrase mRNA and reporter plasmid and transferred back into the recipients. For the optimization of ФC31 integrase mRNA dosage, three concentrations (50, 100 and 200 ng/μL) were tested using parthenogenetic embryos. The optimal dosage turned out to be 200 ng/μL. A penicillin-streptomycin combination mixture was injected intraperitoneally into the recipients to protect against surgical wound infections.

### 4.5. End Point Real-Time and TAIL-PCR

An Long and Accurate (LA) Taq (Takara) DNA polymerase was used in the end-point PCR testing. A typical LA Taq PCR reaction mixture contained 5 μL 10 × LA PCR buffer (Mg^++^ plus), 8 μL dNTP mixture (2.5 mM each), LA Taq polymerase 0.5 μL (5 units/μL), forward and reverse primer 1 μL (20 μM, respectively), 0.5 μg template and PCR-grade water added to 50 μL in total volume. Cycling conditions were 94 °C for 2 min, followed by 30 cycles of 94 °C 30 s, 55 °C 30 s and 72 °C 1 min/kb, followed by final extension for 5 min at 72 °C. A fifth of the volume of the PCR reaction was analyzed on either a 1% or 2% agarose gel and documented using a ChemDOC™ XRS^+^ (Biorad, Berkeley, CA, USA). A SYBR Green I real-time PCR master mix from Toyobo (Osaka, Japan) was used in all quantitative PCR tests performed in a Rotor Gene6000 real-time rotary analyzer (Corbette Lifescience, Eppendorf, Hamburg, Germany). Briefly, the 20 μL reaction mixture included 10 μL 2× master mix, 0.5 μL primer mix (5 μM each), 1 μL template DNA (100 ng for genomic DNA or 5 ng for plasmid DNA) and 8.5 μL PCR-grade water. A two-step amplification protocol was used with the following parameters: a 2-min incubation at 95 °C was used to fully denature the template and activate the Taq DNA polymerase followed by 40 cycles, each of denaturation at 95 °C for 8 s, annealing and extension at 60 °C for 25 s. A final melting temperature analysis from 50 to 99 °C was used to ensure amplicon uniformity. Fluorescence was acquired at annealing and extension steps. All PCR amplifications were performed in triplicate. Relative signal intensities were calculated by the ^ΔΔ^*C*t method with the built-in software RG6000 series 1.7. Copy number of the transgene was calculated using the single-copy *transferrin receptor protein 1 gene (TFRC)* as an internal control with the following formula: Transgene copy number/pig genome = molecules of transgene/molecules of *TFRC* × 2. For the quantitative expression analysis, cDNAs were assessed using the ^ΔΔ^*C*t method and normalized against the reference gene *GAPDH*. TAIL-PCR was conducted essentially as previously described [18]. All primer sequences are listed in Table S1.

### 4.6. Cell Culture

Primary fibroblast cells of the two piglets were isolated from ear tissues when notching. The ear tissues were immediately stored in sterile DPBS with 250 U/mL penicillin and 250 U/mL streptomycin and transported to the laboratory. After rinsing 3 to 5 times in the DPBS and then 70% alcohol, the ear biopsies were cut into pieces of 1–3 mm^3^ in size, and digested in 0.25% trypsin and 0.04% EDTA solution at 4 °C for 1–2 h and then at 39 °C for additional 30–60 min. The treatment was stopped by adding 1 mL of 39 °C pre-warmed DMEM/F12 (GIBCO) culture medium (containing 20% fetal bovine serum, 2 mmol/L glutamine, 100 U/mL penicillin and 100 U/mL streptomycin). The cell density was adjusted to 5 × 10^5^ to 1 × 10^6^ cells/mL with culture medium. The flask was incubated in a 39 °C, saturated humidity, 5% CO_2_ incubator, and the culture medium was replaced every two days.

### 4.7. FACS and ELISA

The FACS analyses were performed using a BD FACS Aria III (BD Biosciences, San Jose, CA, USA). Excitation and emission wavelengths for EGFP were 488 nm and 520 nm, respectively. Briefly, the cells were filtered by a 100-well net and resuspended in DPBS with a total of 2 × 10^5^ cells in each sample for sorting. Average EGFP fluorescence (GFP-A) was measured and the EGFP-positive cell population was collected. The sorted cells were further cultured until they reach 90% confluence and then 1 × 10^6^ cells were harvested and lysed to measure the concentration of EGFP in the cell lysate by an ELISA kit (Abcam, San Francisco, CA, USA). All artwork was created using CorelDRAW Graphics (Corel Corporation, Ottawa, ON, Canada). All data generated or analyzed during this study are included in this manuscript. All materials in this study are available to non-commercial uses.

## 5. Conclusions

Achieving potent and continuous transgene expression in transgenic animals has long been difficult as it is affected by various factors like position effect, transgene orientation, promoter selection, etc. Until now, our understanding of where to insert a gene of interest into the host genome for safe and reliable expression has been limited. Identification of porcine transgene safe harbor in this work has broad application in the creation of transgenic pigs for disease modeling, production of therapeutic proteins and genetic improvement of economic traits. We envision that these novel genomic safe harbors in the pig genome coupled with gene editing technology like CRISPR/Cas9 or recombinase-mediated cassette exchange will accelerate the development and application of transgenic pigs in basic science and agriculture.

## Figures and Tables

**Table 1:** Pig zygote injection summary.

Surrogate	Route	mRNA/DNA Dosage (ng/μL)	Injected/Transferred (%)	Liveborn Founders (%) *	Integrated (%) **	Pseudo attP Site
886	PNI	200/10	32/28 (88)	9 (32)	2 (22)	0
1023	PNI	200/10	33/25 (76)	8 (32)	2 (25)	1
654	PNI	200/10	28/20 (71)	8 (40)	3 (38)	1
982	ICI	200/10	29/24 (83)	7 (29)	0 (0)	N/A
10,665	ICI	200/10	30/23 (77)	return	N/A	N/A

PNI: pronuclear injection; ICI: intracytoplasmic injection; *: of embryos transferred; **: of liveborn founders; N/A: not available.

## Supplementary Materials

The following are available online at www.mdpi.com/1422-0067/19/1/149/s1.
